# A Novel Fracturing Fluid Based on Functionally Modified Nano-Silica-Enhanced Hydroxypropyl Guar Gel

**DOI:** 10.3390/gels10060369

**Published:** 2024-05-27

**Authors:** Feifei Huang, Yun Bai, Xiaoyu Gu, Shaofei Kang, Yandong Yang, Kai Wang

**Affiliations:** 1 School of Petroleum Engineering and Environmental Engineering, Yan’an University, Yan’an 716000, China; 2 School of Petroleum Engineering, China University of Petroleum (East China), Qingdao 266555, China; 3 School of Petroleum Engineering, Xi’an Shiyou University, Xi’an 710065, China

**Keywords:** low-permeability reservoirs, fracturing fluid, formation damage, nano-silica, hydroxypropyl guar, property evaluation

## Abstract

Considering the damage caused by conventional fracturing fluid in low-permeability reservoirs, a novel fracturing fluid (FNG) combining hydroxypropyl guar (HPG) and functionally modified nano-silica (FMNS) was prepared. The properties of heat/shear resistance, rheological property, proppant transportation, and formation damage were evaluated with systematic experiments. The results showed that the viscosities of FNG before and after the heat/resistance were 1323 mPa·s and 463 mPa·s, respectively, while that of conventional HPG gel was 350 mPa·s. FNG is a pseudoplastic strong gel with a yield stress of 12.9 Pa, a flow behavior index of 0.54, an elastic modulus of 16.2 Pa, and a viscous modulus of 6.2 Pa. As the proportions of proppant mass in further sections transported with FNG were higher than those transported with HPG gel, FNG could transport the proppant better than HPG gel at high temperatures. Because of the amphiphilic characteristics of FMNS, the surface/interface properties were improved by the FNG filtrate, resulting in a lower oil permeability loss rate of 10 percentage points in the matrix than with the filtrated HPG gel. Due to the considerable residual gel in broken HPG gel, the retained conductivity damaged with broken FNG was 9.5 percentage points higher than that damaged with broken HPG gel. FNG shows good potential for reducing formation damage during fracturing in low-permeability reservoirs in China.

## 1. Introduction

As the oil production from conventional reservoirs is constantly in decline, the low-permeability oil reservoir is an important succeeding resource for the exploitation of hydrocarbon, especially in China [1,2,3]. The reserve of low-permeability reservoirs is about 4495 × 108 t, accounting for 48% of the total global oil reserves [4]. Low-permeability reservoirs are low in pressure and have low original oil in place (OOIP), resulting in extremely low oil supply capacity. Therefore, most of the oil wells in low-permeability reservoirs are put into production with fracturing [3,5]. However, due to the damage of the fracturing fluid, not all of the wells can increase production from fracturing [6]. Some wells even show negligible production after fracturing [7].

Hydroxypropyl guar (HPG) gel, the most popular fracturing fluid, can clog the pores and throats in the matrix, and inhibit the conductivity of fractures due to considerable insoluble residue and residual gel [8]. Meanwhile, it reduces the permeability of the oil phase by the water locking effect due to the high interfacial tension (IFT) between the oil and the filtrate [9,10]. Jianchun Guo et al. [7] revealed that galactose in the side chain is dissociated faster than mannose in the main chain during the early stage of guar gum gel breaking caused by the oxidizing agent such as ammonium persulfate. It leads to a substantial increase in the M/G (D-mannuronate to L-guluronate) ratio, a decrease in molecular solubility, and a considerable residue after gel breaking. Xuejiao Li et al. [3] studied the microscopic damage mechanism of fracturing fluids to low-permeability sandstone with nuclear magnetic resonance and pointed out that water locking caused by hydroxypropyl guar brings a matrix permeability loss ratio as more as 23.26%.

In view of the above damages, slickwater, viscoelastic surfactant (VES), and foam fluid are widely taken into consideration as low-damage fracturing fluids for the small amounts of insoluble residue and residual gel [9,11,12,13]. However, slickwater is just used as the prepad followed by the proppant-transporting fluid during fracturing such as guar gel because of the poor proppant transport performance and narrow fracture widths caused by the low viscosity [14]. Meanwhile, the poor heat resistance [13] and high cost according to the immense consumption (the dosage of surfactant is usually 3–5 wt%) restrict the extensive application of VES fracturing fluids [15]. Furthermore, besides the sand concentration limits, the potential safety concerns of pumping foam at high pressure, the operational issues related to handing gas onsite, and the increasing pumping capacity caused by the associated friction losses, foam fluids are very expensive to truck the gas in most regions without ready access to the gases [11].

Iler [16] pointed out that adding nanoparticles could lead to the adsorption of polymer chains on the nanoparticle surface, resulting in the viscosity enhancement of polymer gel at high temperature [17]. It indicates that the dosage of polymer required by the viscosity retention of fracturing fluid could be decreased with the presence of nano-silica. As the most widely used nanoparticle, nano-silica demonstrates great potential in improving rheology and increasing crude oil production [18,19,20]. Nevertheless, nano-silica naturally tends to adhere to each other and further develops large-dimension clusters due to the high surface area to volume and Van der Waals’ force. Meanwhile, the brine in the oil reservoirs weakens the electrostatic repulsion between nano-silica clusters [21,22]. As a result, the formed clusters are detained in the small and narrow pore throats of the matrix and decrease the permeability of the fractured oil reservoirs [23,24]. Fortunately, the dispersion enhancement and amphiphilic characteristics of nano-silica were realized via functional modification in the previous study [25]. This shows great potential in the application of the fracturing fluid to decrease the dosage of polymer, thus decreasing the damage to the formation caused by residue and residual gel in the fracturing fluid. Furthermore, the damage caused by water locking is also promisingly decreased for the IFT reduction based on amphiphilic characteristics [12,26,27,28,29,30].

In this paper, a novel fracturing fluid FNG was prepared by combining the functionally modified nano-silica (FMNS) and hydroxypropyl guar. Then, the properties of heat/shear resistance, rheology, proppant transportation, and formation damage were evaluated systematically.

## 2. Results and Discussion

### 2.1. Heat/Shear Resistance

**Figure 1:**
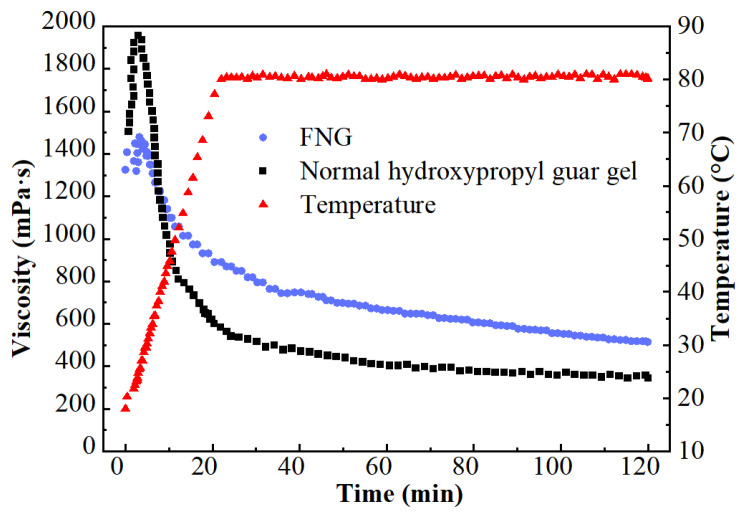
Curves of the heat/shear resistance test.

The results of the heat/shear resistance are shown in Figure 1. The viscosity at the initial HPG gel (1507 mPa·s) was much higher than that of FNG (1323 mPa·s) for there was a higher dosage of polymer in HPG gel. However, the viscosity of HPG gel decreased rapidly after a brief rise as the temperature rose, while the viscosity of FNG decreased laxly. In the end, the retained viscosity of FNG was 463 mPa·s, which was significantly higher than that of HPG gel (350 mPa·s).

**Figure 2:**
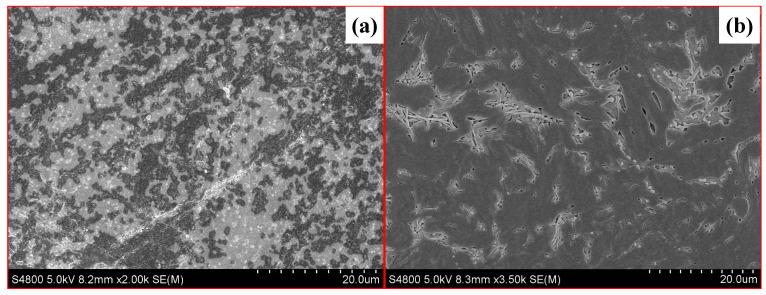
SEM photos of FNG (**a**) and HPG gel (**b**).

To reveal the microstructures of the difference between these two fracturing fluids, the samples after the test were characterized by high-resolution mass spectrometer scanning electron microscopy (SEM, S4800, Hitachi, Tokyo, Japan). As the SEM photos show in Figure 2, the polymer in FNG formed a larger and more robust spatial structure, resulting in a larger hydrodynamic radius.

**Figure 3:**
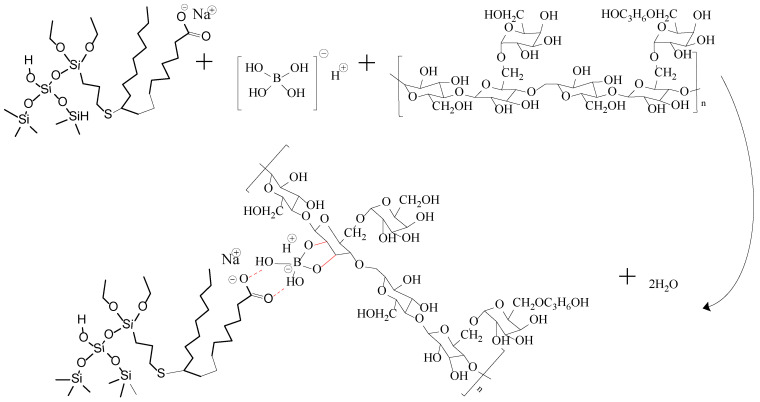
Mechanism diagram of dual cross-linking in FNG.

**Figure 4:**
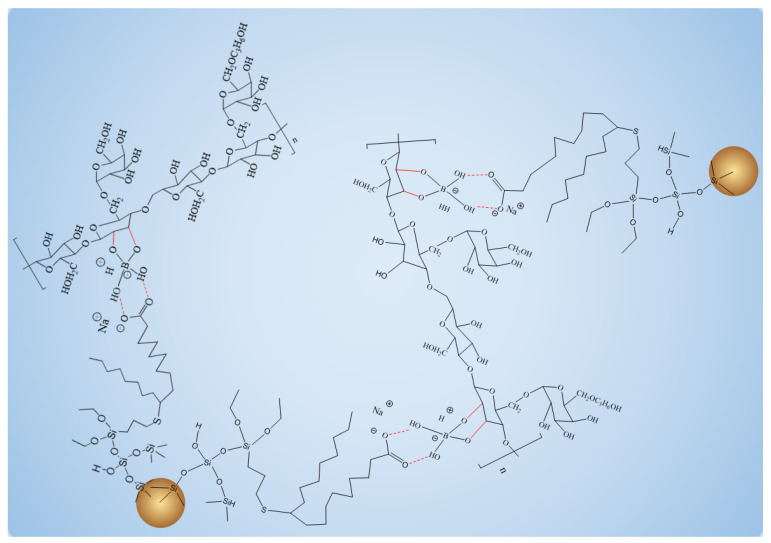
Schematic diagram of overall structure enhanced by FMNS.

When Na_2_B_4_O_7_ is hydrolyzed, boronate ions are generated in the route of Equations (1) and (2). As shown in Figure 3 and Figure 4, borate ions connected to hydroxypropyl guar gum and carboxyl groups on the modified nano-silica surface through hydroxyl dehydration condensation and hydrogen bonding, respectively, resulting in the dual-level cross-linking structure of nano-silica–borate ions and borate ions–hydroxypropyl guar. Nanoparticles are less affected by molecular motion under shear at high temperatures, thus promoting the stability of the overall structure under the effect of heat/shear [31,32].

(1) $${\mathrm{Na}}_{2}B_{4}O_{7}+7H_{2}O\to 2{\mathrm{Na}}^{+}+4B{\left(\mathrm{OH}\right)}_{3}+2{\mathrm{OH}}^{-}$$

(2) $$B{\left(\mathrm{OH}\right)}_{3}+H_{2}O⇄B{{\left(\mathrm{OH}\right)}_{4}}^{-}+H^{+}$$

### 2.2. Rheology Properties

**Figure 5:**
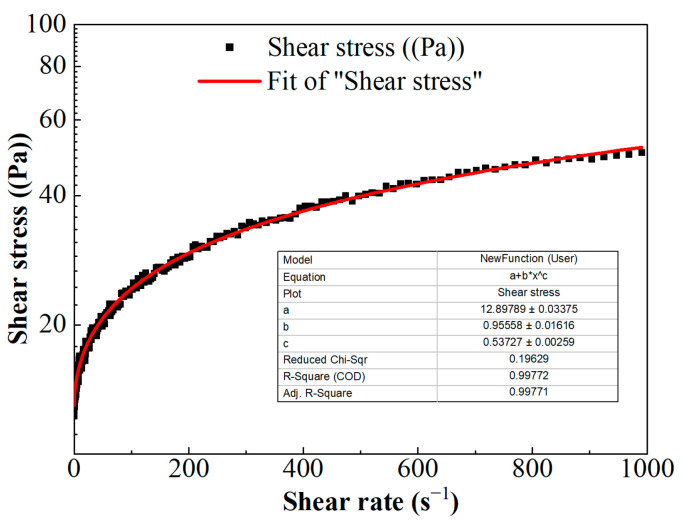
The curve of shear stress versus shear rate.

The scatter of shear stress versus shear rate is shown in Figure 5. The nonlinear relationship between shear stress and shear rate indicates that FNG is a typical non-Newton fluid. According to the relevant theory, the non-Newton fluid can be divided into pseudoplastic fluid, dilatant fluid, Bingham plastic fluid, and Bingham pseudoplastic fluid. The viscosity of the fluid decreases with the increase in shear rate in pseudoplastic fluid, the viscosity of the fluid increases with the increase in shear rate in dilatant fluid, and the fluid could only flow under high stress with linear fluidity in Bingham plastic fluid but with nonlinear fluidity in Bingham pseudoplastic fluid.

The relationship of shear stress versus shear rate in Figure 5 could be described with the Herschel–Bulkley equation [33]:

(3) $$\tau =\tau_{0}+k\times \gamma^{n}$$

where *τ* is the shear stress, Pa; *γ* is the shear rate, s^−1^; *τ*_0_ is the yield stress, Pa; *k* is the consistency coefficient, Pa·s^n^; and *n* is the flow behavior index, dimensionless. These parameters were obtained via the fitting curve in Figure 5 and as shown in Equation (4).

(4) $$\tau =12.90+0.96\times \gamma^{0.54}$$

(5) μ=τ/γ×1000

where *μ* is the viscosity, mPa·s.

**Figure 6:**
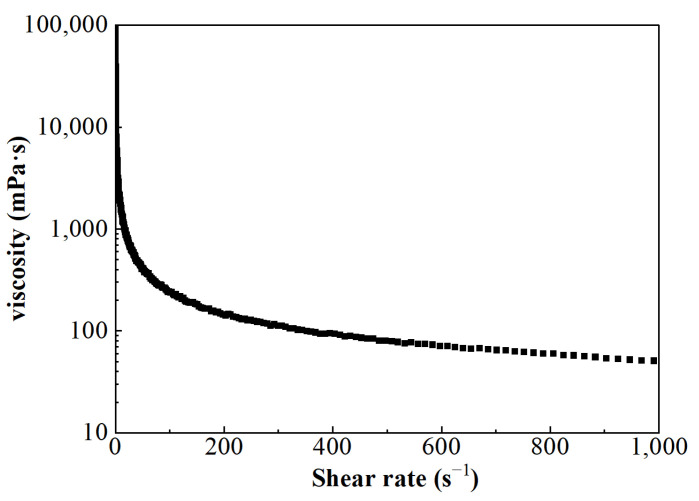
The curve of viscosity–shear rate.

The viscosity of FNG under different shear rates can be obtained according to Equation (5), and the result is shown in Figure 6. Besides the fact that the flow behavior index *n* was equal to 0.54, which satisfied the condition of 0 < *n* < 1, the calculated viscosity under low shear rates was very high and the viscosity decreased as the shear rate increased. These show that FNG is a Bingham pseudoplastic fluid with a yield stress of 12.9 Pa.

**Figure 7:**
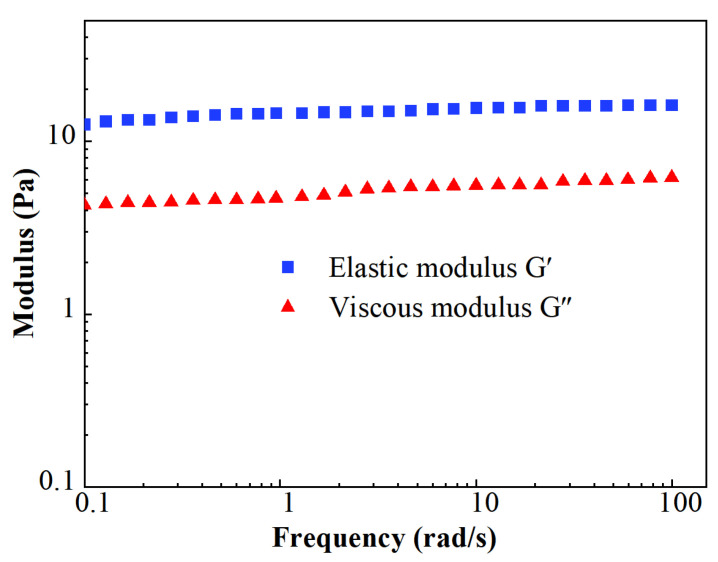
Scatters of viscoelastic modulus.

The results of the modulus measurements are shown in Figure 7. The stable level of elastic moduli *G*′ was about 16.2 Pa, while the value of viscous moduli *G*″ was about 6.2 Pa. The value of *G*′ remained higher than *G*″ during the whole test of the viscoelastic modulus. It indicates that FNG is a typical elastic fluid.

### 2.3. Proppant Transportation

**Figure 8:**
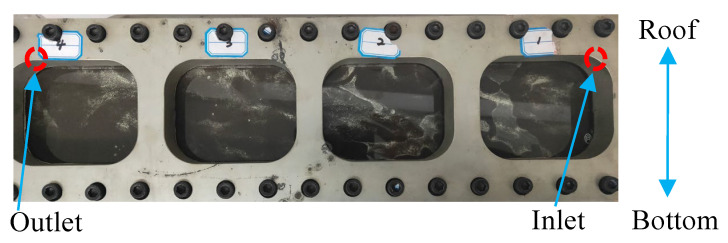
Picture of the proppant transportation.

The photograph of proppant settlement in the crack after the injection of fracturing fluid containing proppant is shown in Figure 8. It could be found in Figure 8 that the proppant aggregated near the entrance more than in the other areas. In addition, the proppant was distributed more around the bottom than the roof along the crack; even the visual differences in proppant distribution between sections were not significant.

**Figure 9:**
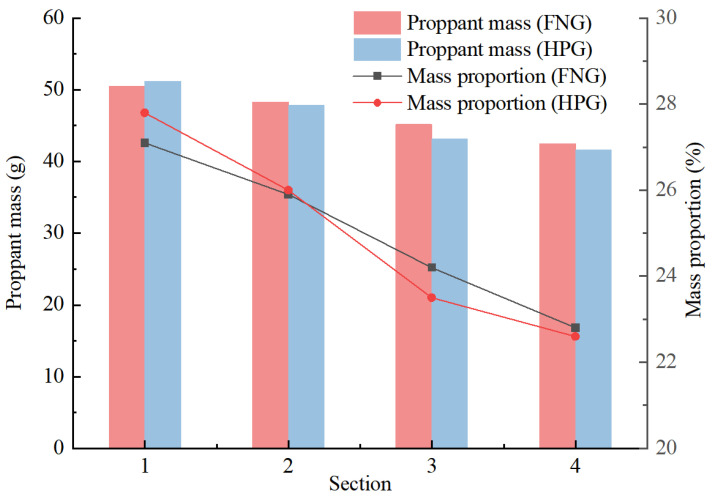
Statistical chart of proppant transportation.

The results of the statistics are shown in Figure 9. The mass proportions along the crack reflected the rate of the proppant subsiding during the injection. The proportions in Section 1 (inlet) and Section 2 of HNG were lower than those of HPG gel, and the proportions in Section 3 and Section 4 (outlet) were higher than those of HPG gel. It indicates that in accordance with the heat/shear resistance, there were more proppants transported to a long distance by FNG. That is beneficial to the liquid flowing in the crack after fracturing.

### 2.4. Formation Damage

The parameters of the natural cores used in the formation damage test and the results of the measurements are shown in Table 1. There were similar water permeability losses caused by FNG and HPG gel filtrate, while the oil permeability loss caused by FNG filtrate was much less than that caused by HPG gel filtrate. It is beneficial for the oil to flow into the wellbore after fracturing.

The permeability of the oil phase in the presence of residual water is affected by the interfacial parameters such as the contact angle (CA) and the interfacial tension (IFT) between oil and fracturing fluid filtrates. To analyze the difference in the oil permeability damage between FNG filtrate and HPG gel filtrate, the CA of deionized water (DI water) on the core surface and the IFT between oil and fracturing fluid filtrates were tested.

The tests of the CAs were carried out with smooth natural core slices. Initially, three different slices were merged in formation water, FNG filtrate, and HPG gel filtrate for 24 h, respectively. Then, the DI water was dripped onto the three slices merged in the kerosene and the CAs could be obtained with the optical contact angle tester. To test the IFTs, the formation water, FNG filtrate, and HPG gel filtrate were filled into the cells first. The simulated crude oil was then injected into the sample cells, and the IFTs could be obtained with the interface tension meter.

**Figure 10:**
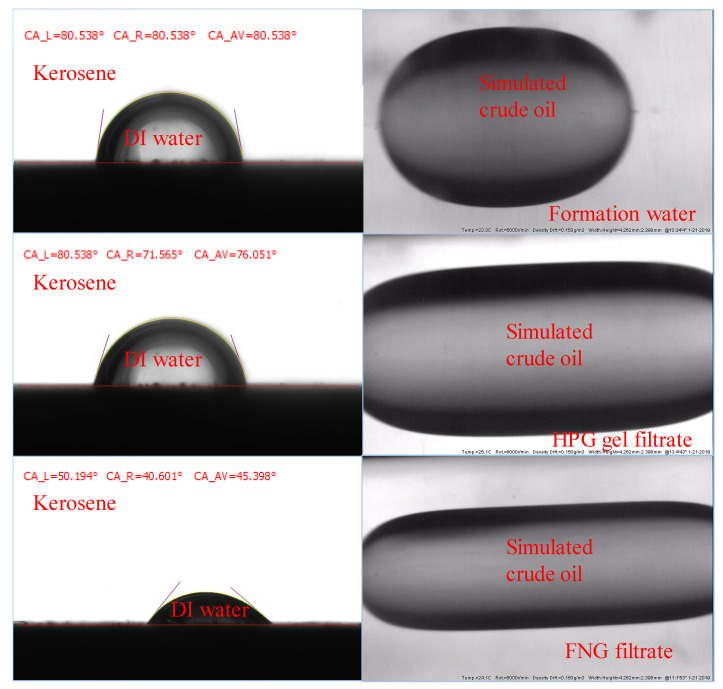
Images during the measurements of the CAs and IFTs.

The results are shown in Figure 10, where the core slices were merged in formation water, HPG gel filtrate, and FNG filtrate in the order from top to bottom. It could be found that the CA of the slice merged in FNG filtrate was about 45°, while the CAs of the slices merged in formation water and HPG filtrate were about 70–80°. The IFT between FNG filtrate and simulated crude oil was 1.23 mN/m. The IFT between formation water and crude oil was 15.38 mN/m, and the IFT between HPG filtrate and crude oil was 6.52 mN/m. Because of the amphiphilic characteristics of FMNS, the CA and IFT measured with FNG were much lower than those measured with HPG gel filtrate, resulting in a lower resistance for oil flowing in the presence of residual water. It is the reason that the permeability loss of oil caused by FNG filtrate was much lower than that caused by HPG gel filtrate in Table 1.

**Figure 11:**
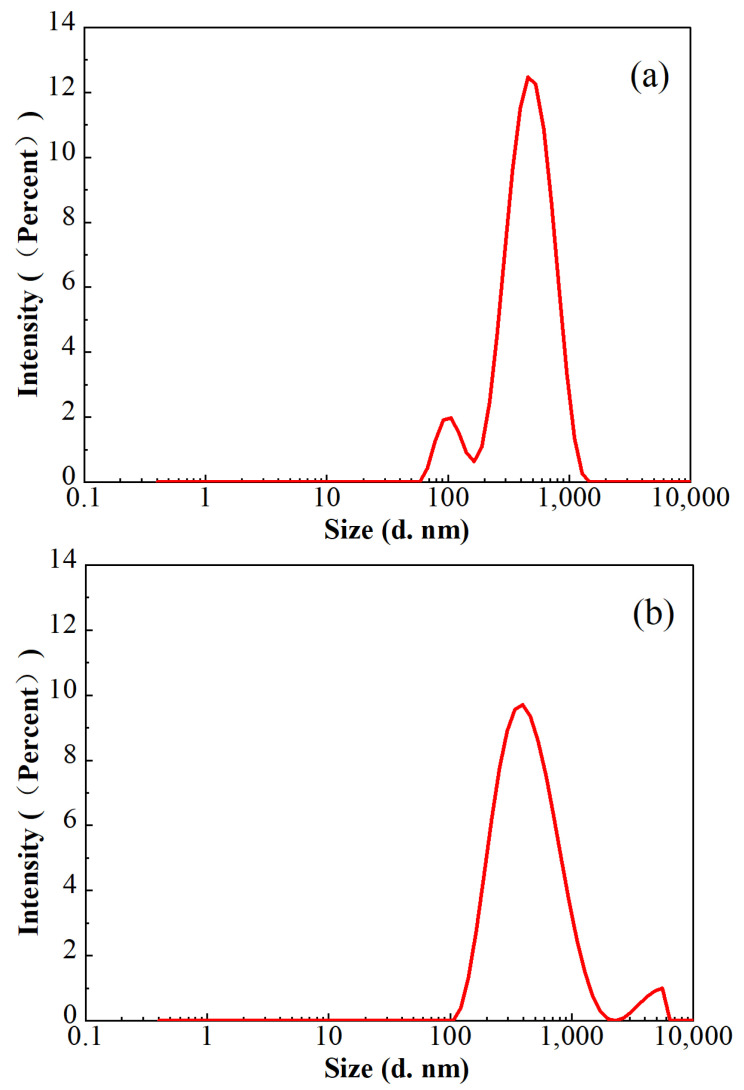
Particle size distributions in the broken FNG (**a**) and broken HPG gel (**b**).

In consideration of the lower polymer dosage in FNG, the sizes of the residue in the broken fracturing fluids were analyzed with a Malvern laser particle size analyzer at 25 °C, and the results are shown in Figure 11. The average residue sizes in both of these two broken fracturing fluids were approximately 360 nm, and the proportion of the residue sized from 200 nm to 700 nm was higher than 90%. However, a peak at 108 nm with an intensity of 8.6% was found in broken FNG, while a peak at 4522 nm with an intensity of 3.5% was found in broken HPG. The size of 108 nm is slightly higher than the size of 58 nm, which corresponds to the peak of the FMNS dimension distribution. The size of the general polymer residue is much smaller than 4522 nm, so the peak at 4522 nm should correspond to the residual gel that had not broken completely due to the high dosage of polymer and cross-linker. Therefore, although the addition of FMNS brings some tiny residue in the broken FNG, it eliminates the much larger residual gel via decreasing the dosage of polymer and cross-linker, which weakens the damage significantly.

The results of the conductivity retainment are shown in Table 2. The conductivity retainment rate contaminated with broken FNG was nearly 10% higher than that contaminated with broken HPG gel, which is consistent with the measurement of the residue size shown in Figure 11.

The properties of FNG were compared with the previously published low-damage fracturing fluid, and the results are shown in Table 3. Because of the different focuses of published works, the information on the previously published low-damage fracturing fluid was not complete. Even so, it could be found that FNG shows the highest viscosity after the heat/shear measurement, and the permeability loss rate of FNG is lower than nanoparticle-enhanced VES and tertiary cross-linked guar.

## 3. Conclusions

Aiming at resolving the formation damage caused by the residue and residual gel of conventional fracturing fluid, a novel fracturing fluid FNG combining the functionally modified nano-silica and low-dosage hydroxypropyl guar was prepared. Furthermore, systematic experiments were carried out to evaluate its properties. The following conclusions were drawn from this work:

1. Due to the dual-level cross-linking structure in FNG and the small influence of high temperature on nano-silica molecular motion, the retained viscosity of FNG was 463 mPa·s after the heat/shear resistance measurement, while that of HPG gel was about 350 mPa·s.
2. FNG is a pseudoplastic strong gel with a yield stress of 12.9 Pa, a flow behavior index of 0.54, an elastic modulus of 16.2 Pa, and a viscous modulus of 6.2 Pa.
3. In accordance with the heat/shear resistance, FNG could realize better proppant transportation under high temperatures than HPG gel.
4. As the lower water CA and lower IFT entered the FNG, the damage on oil permeability in the matrix caused by FNG filtrate was 10 percentage points lower than that caused by HPG gel filtrate. Meanwhile, because of the considerable residual gel in the broken HPG gel, the conductivity retainment contaminated with broken HPG gel was 9.5 percentage points lower than that with broken FNG.

## 4. Materials and Methods

### 4.1. Material and Instruments

**Figure 12:**
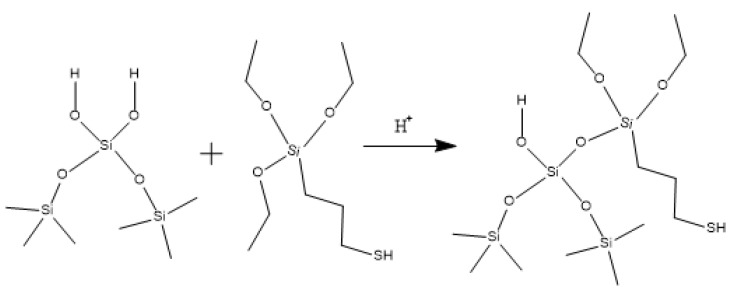
Synthesis route of mercapto-terminated nano-silica.

**Figure 13:**
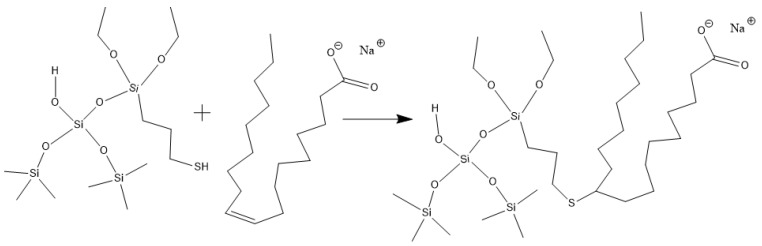
Route of integrating sodium oleate to the mercapto-terminated nano-silica.

Hydroxypropyl guar, an industrial product, was purchased from Shandong Guangpu Biotechnology Co., Ltd. located in Zibo, China. Sodium tetraborate (Na_2_B_4_O_7_, net content 99%), ammonium persulfate (APS, H_8_N_2_O_8_S_2_, AR), and sodium hydrogen carbonate (NaHCO_3_, AR) were purchased from Shanghai Aladdin Biochemical Technology Co., Ltd. located in Shanghai, China. Proppant (mesh size 30/50) was obtained from Tianhong proppants Co., Ltd. located in Yangquan, China. The deionized water (DI water) was prepared in the lab, while natural core samples (the average permeability and porosity were about 0.9 × 10^−3^ μm^2^ and 12.8%, respectively), formation water (CaCl_2_, salinity 17,000 mg/L, density 1.01 g/cm^3^, viscosity 1.38 mPa·s at 18 °C), and crude oil were acquired from Xiasiwan Chang 8 tight oil formation, Ordos Basin, China. The simulated crude oil (density 0.83 g/cm^3^, viscosity 2.88 mPa·s at 18 °C) was prepared by mixing the kerosene (obtained from Jinan Xinquan Chemical Technology Co., Ltd. located in Jinan, China) and the crude oil at a volume ratio of 3:1. The functionally modified nano-silica FMNS was prepared via two major procedures in the lab. The first procedure was to synthesize mercapto-terminated nano-silica. The second procedure was to integrate sodium oleate into the mercapto-terminated nano silica via the thiol-ene “click” reaction [38]. The particle dimension distribution of the modified nano-silica presents a single peak at 58 nm, while the original nano-silica shows two peaks at 32 nm (less than 5%) and 120 nm. The details of the modified nano-silica preparation can be found in the previous work [25]. Taking nano-silica with germinal hydroxyl groups on the surface as an example, the reaction mechanism diagram is shown in Figure 12 and Figure 13.

MCR302 rheometer was from Anton Paar, Graz, Austria. TX-500C Interface tension meter was from BOWING INDUSTRY CORP, Everett, WA, USA. The SL200KB Optical Contact Angle Tester was from KINO Industry Co. Ltd., New York, NY, USA. Proppant transportation tester and core displacement system were from Nantong Huaxing Petroleum Instrument Co., Ltd., Nantong, China.

### 4.2. Preparation of the Fracturing Fluids

Initially, 4 g hydroxypropyl guar was gradually added into the beaker marked A containing 1000 mL DI water at a stirring rate of 50 rmp, while 6 g hydroxypropyl guar was added into beaker B containing 1000 mL DI water at the same stirring rate. The guar solutions were kept under the static condition at room temperature for 4 h. Secondly, 1 g FMNS, 4 g Na_2_B_4_O_7_, 0.05 g APS, and 1 g NaHCO_3_ were added into beaker C containing 1000 mL DI water under stirring, while 6 g Na_2_B_4_O_7_, 0.07 g APS, and 1 g NaHCO_3_ were added into beaker D containing 1000 mL water. Finally, the liquids in beaker A and beaker C were mixed under stirring to obtain the novel fracturing fluid FNG with 0.2 wt% hydroxypropyl guar, while the liquids in beaker B and beaker D were mixed under stirring to obtain HPG gel with the hydroxypropyl guar dosage of 0.3 wt% as the control sample.

### 4.3. Evaluation of the Properties

#### 4.3.1. Heat/Shear Resistance

The fracturing fluid is sheared seriously during the extension of the fractures in the formation, and the temperature in the formation is usually high. Good heat/shear resistance is necessary for the fracturing fluid to work underground effectively. The heat/shear resistance of FNG and HPG gel were tested with the rheometer. The temperature ranged from room temperature (at 18 °C) to 80 °C with a heating rate of 3 °C /min, while the shear rate was kept at 170 s^−1^ constantly according to the Chinese standard of SY/T 5107-2016 [33].

#### 4.3.2. Rheological Property

The rheological property shows a significant impact on the load of the pumps, which is usually very high during the fracturing operation. At the same time, it provides valuable parameters and theoretical guidelines for the design of the operation and the optimization of the fracturing fluids, such as in numerical simulation. The rheological measurements were carried out with the rheometer. The viscoelastic modulus of FNG was tested under frequency scanning in the range of 0.1–100 rad/s. The shear stress–shear rate curve was tested to ensure the rheological parameters under the shear rate ranging from 0.01 to 1000 s^−1^. The details of the tests referred to the Chinese standard of SY/T 6296-2013 [39]. Unless specified, all the experiments were carried out at room temperature, and the same below.

#### 4.3.3. Proppant Transportation

The fundamental objective of fracturing is to promote the seepage of the liquid in the formation through the conductivity of artificial fracture. The fractures would close after the fracturing operation, so the conductivity of the closed fractures mainly rely on the propping of the proppant. Therefore, proppant transportation is critical to the fracturing operation, which is necessary for the transportation of more proppant into the depths of the cracks [40]. The capacity of proppant transportation was compared between FNG and HPG gel. The fracturing fluids with a proppant concentration of 30 wt% were injected into a simulated crack under 80 °C at a rate of 5 mL/min for 1.5 h, and the excess fracturing fluid could overflow from the outlet. The width, height, and length of the crack were 0.15 cm, 20 cm, and 100 cm, respectively. To compare the settlement of the proppant during transportation, the crack was divided into four sections with an observing window on each section. The masses of left proppant belonging to each section were selected, dried, and weighted in sequence after the injection. The proportion of the proppant mass could be obtained according to Equation (6):

(6) $$\eta_{i}=\frac{m_{i}}{\sum_{i=1}^{4}m_{i}}\times 100\%$$

where *η_i_* is the proportion of the proppant mass in section *i* of the crack, %; *m_i_* is the mass of the proppant in section *i* of the crack, g; and *i* is the number of the divided sections in the crack, dimensionless.

#### 4.3.4. Formation Damage Evaluations

There is a considerable proportion of fracturing fluid that could not flow back from the formation, especially in the low-permeability reservoirs due to the narrow pores and throat. Furthermore, the residual gel and residue left in the cracks also could not be ignored after the filtration of the fracturing fluid. All these would cause inevitable formation damage such as matrix permeability losses and crack conductivity losses, which run counter to the objective of the fracturing operation. The matrix permeability losses caused by FNG and HPG gel were tested with natural core samples, while the retainment conductivity was measured with the cracked natural core samples. Taking the influence of filtrates on the surface properties into consideration, the permeability loss of formation water and simulated crude oil were measured, respectively, while the conductivities were just tested with formation water. According to the Chinese standard of SY/T 5107-2016 [33], the measurement of the permeability loss of the formation water was carried out as follows:

(1) The core was saturated with formation water, and the initial permeability could be obtained when the water flooding became stable.
(2) The fracturing fluid filtrate was injected into the core and retained in the core for 2 h.
(3) The core was flooded with the formation water again to obtain the damaged permeability of the formation water.

The measurement of the oil permeability loss was carried out as follows:

(1) The core was saturated with formation water.
(2) The core saturated with formation water was flooded with the oil. The initial oil permeability in the presence of residual water could be obtained when the oil flooding became stable and there was no more water flow out of the core.
(3) The fracturing fluid filtrate was injected into the core and retained in the core for 2 h.
(4) The core was flooded with the oil again to obtain the damaged permeability of the oil.

## Figures and Tables

**Table 1:** Statistics of the matrix permeability losses.

Cores	Filtrate	Medium	Initial Permeability (×10^−3^ μm^2^)	Damaged Permeability (×10^−3^ μm^2^)	Permeability Loss Rate (%)
A-1	FNG	Formation water	0.95	0.74	22.1
B-1		Simulated crude oil	0.87	0.76	12.6
A-2	HPG gel	Formation water	0.96	0.76	20.8
B-2		Simulated crude oil	0.85	0.66	22.4

**Table 2:** Statistics of the conductivity retainment.

Cores	Contaminant	Initial Conductivity (×10^−3^ μm^2^·mm)	Damaged Permeability (×10^−3^ μm^2^·mm)	Conductivity Retainment Rate (%)
A-3	Broken FNG	2350	1895	80.6
B-3	Broken HPG gel	2865	2038	71.1

**Table 3:** Comparison between FNG and published low-damage fracturing fluids.

Results	FNG	NESF [1]	Supramolecular Fluid [34,35]	Nanoparticle Enhanced VES [36]	Tertiary Cross-Linked Guar [37]
Viscosity (mPa·s)	463 (80 °C)	154 (80 °C)	71 (120 °C)	90 (45 °C)	78 (80 °C)
Permeability loss rate (%)	12.6	9.4	12.2	31.1	13.5
Conductivity retainment (%)	80.6	95.11	92		

## Data Availability

All data and materials are available upon request from the corresponding author.

## Nomenclature

OOIP	original oil in place
HPG	hydroxypropyl guar
M/G ratio	ratio of D-mannuronate to L-guluronate
IFT	interfacial tension
VES	viscoelastic surfactant
FMNS	functionally modified nano-silica
DI water	deionized water
*μ*	viscosity, mPa·s
*τ*	shear stress, Pa
*γ*	shear rate, s^−1^
*τ*_0_	yield stress, Pa
*k*	the consistency coefficient, Pa·s^n^
*n*	the flow behavior index, dimensionless
*G*′	elastic moduli
*G*″	viscous moduli
CA	contact angle
*η_i_*	the proportion of the proppant mass in section *i* of the crack, %
*m_i_*	mass of the proppant in section *i* of the crack, g
*i*	the number of the divided section in the crack, dimensionless
